# Pelvic posture and kinematics in femoroacetabular impingement: a systematic review

**DOI:** 10.1007/s10195-016-0439-2

**Published:** 2017-02-01

**Authors:** Luca Pierannunzii

**Affiliations:** Gaetano Pini Orthopedic Institute, P.zza C. Ferrari, 1, 20122 Milan, Italy

**Keywords:** Femoroacetabular impingement, Pelvic kinematics, Pelvic posture, Gait analysis, Pelvic tilt

## Abstract

**Background:**

Pelvic posture and kinematics influence acetabular orientation and are therefore expected to be involved in the pathomechanics of femoroacetabular impingement (FAI). This systematic review aims to determine whether FAI patients show pelvic postures or patterns of motion contributing to impingement or, conversely, develop compensatory postures and patterns of motion preventing it.

**Materials and methods:**

PubMed/MEDLINE, Embase, Google Scholar and the Cochrane Library were systematically searched to find all the studies that measured pelvic positional and/or kinematic data in humans (patients or cadaveric specimens) affected by FAI.

**Results:**

Twelve items were selected and grouped according to the main field of investigation. No quantitative data synthesis was allowed due to methodological heterogeneity. Pelvic posture and kinematics seem to play a relevant role in FAI. The patients, especially if symptomatic, show a paradoxical lack of pelvic back tilt in standing hip flexions, i.e., in squatting, that enhances femoroacetabular engagement. Such an aberrant pattern might depend on a lower pelvic incidence. On the contrary, active hip flexion in decubitus elicits a compensatory, more pronounced back tilt to facilitate hip flexion without impingement. Stair climbing shows a compensatory pattern of augmented pelvic axial rotation and augmented peak forward tilt to reduce painful hip motions, namely internal rotation and extension.

**Conclusion:**

In FAI patients, pelvic posture and kinematics are sometimes an expression of compensatory mechanisms developed to reduce pain and discomfort, and sometimes an expression of paradoxical responses that further enhance the impingement pathomechanism.

**Level of evidence:**

IV.

**Electronic supplementary material:**

The online version of this article (doi:10.1007/s10195-016-0439-2) contains supplementary material, which is available to authorized users.

## Introduction 

Femoroacetabular impingement (FAI) is a dynamic conflict between the proximal femur head–neck junction and the acetabular rim that may cause progressive chondro-labral damage leading to secondary hip osteoarthritis [1]. Such a dynamic abutment depends not only on the pathoanatomy of proximal femur and acetabular rim, but also on the pathomechanics of the hip joint. While femoral motion, especially flexion and internal rotation, was immediately considered responsible for femoroacetabular engagement [2], functional acetabular orientation (as a consequence of pelvic posture and motion) was only recently considered.

### Pelvic posture

The spinopelvic balance is the condition that allows humans to acquire verticality in the most economical fashion; lumbar lordosis, anterior pelvic tilt and hip extension contribute equally to bipedalism saving the maximum amount of energy [3].

These adaptations aim synergically at placing the C7 PL (the plumb line passing through the centroid of C7 vertebral body) as close as possible to the posterior edge of the sacral plate on the sagittal plane [4]. In a well-balanced spine, the C7 PL passes through or slightly behind this reference, but in a progressively unbalanced spine it passes more anteriorly. The more unbalanced the spine is, the more costly is the verticality, as posterior trunk muscles have to counterbalance the gravity force momentum trying to bend the upper body forward.

**Fig. 1:**
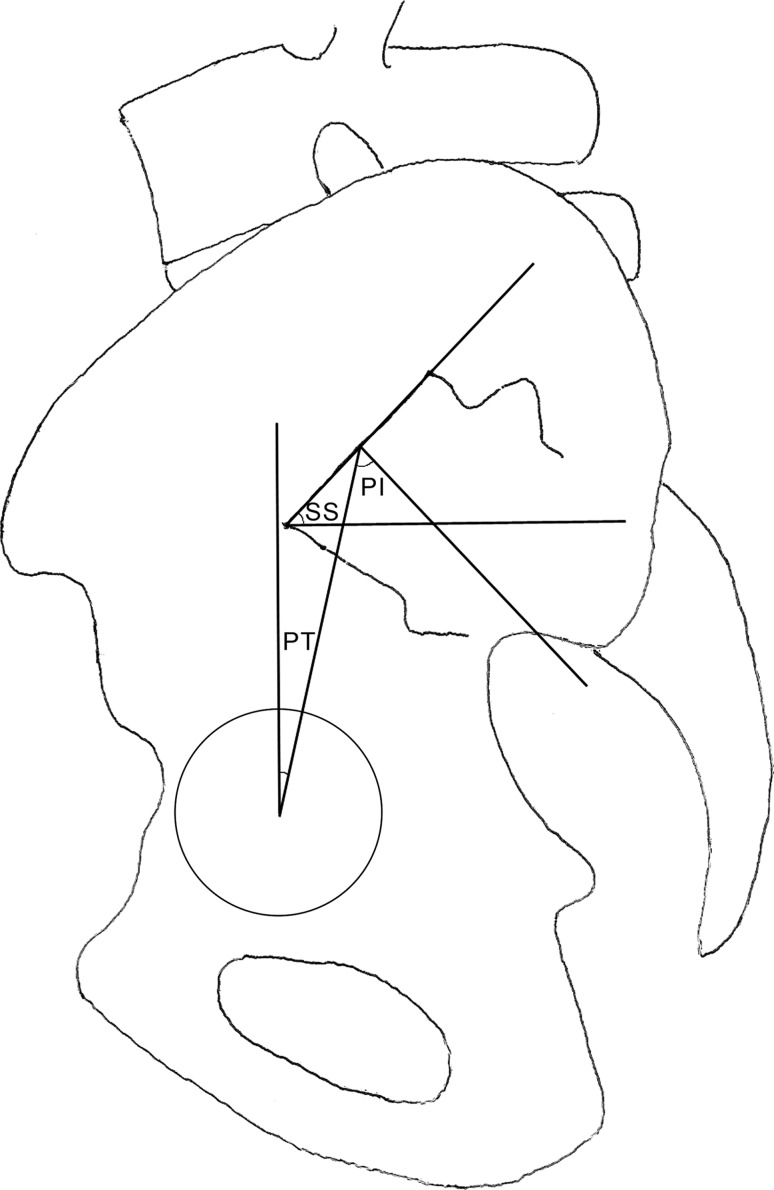
Main pelvic parameters *PI* pelvic incidence, *PT* pelvic tilt, *SS* sacral slope

Thus, our body tries to compensate any local sagittal imbalance through adaptation of the anatomical region immediately distal, sequentially involving lumbar hyperextension, pelvic back tilt, knee flexion and lastly ankle extension, until the gravity line is moved back to the feet. Of these adaptations, pelvic back tilt clearly influences the hip function; it occurs around the bicoxofemoral axis and is fundamentally limited by pelvic incidence (PI) and hip extension. PI, first described by Duval-Beaupere et al. [5–7], is a morphological parameter (i.e., independent of pelvic orientation) that measures the available angular posterior displacement of the sacral plate with respect to the femoral heads. The wider the PI, the greater is the amount of pelvic back tilt theoretically available. It is calculated as the sagittal angle between the line joining the midpoint of the sacral plate and the center of the femoral head (or the bicoxofemoral axis midpoint) and the line perpendicular to the sacral plate (Fig. 1). Sagittal orientation of the pelvis is described by two positional interdependent parameters—pelvic tilt (PT) and sacral slope (SS). SS represent the sagittal acute angle between the tranverse plane and the plane tangent to the sacral plate; the higher the SS, the steeper the basis of the lumbar spine, conditioning a higher degree of lumbar lordosis. PT is the sagittal acute angle between the vertical line and the line joining the center of the femoral head (or the bicoxofemoral axis midpoint) to the anteroposterior (AP) midpoint of the sacral plate (Fig. 1). In other words, PI measures the maximum available posterior displacement of the sacral base, while PT measures the actual displacement. The geometrical relationship between these three pelvic parameters is: PI = PT + SS; reference values of healthy subjects are 55.1° ± 9° for PI, 12° ± 6.4° for PT, 41.2° ± 7° for SS (in standing posture) [5].

Since the acetabular opening is oblique with respect to all the reference planes, the PT (that measures the pure sagittal rotation) dramatically changes the socket orientation, potentially contributing to or protecting from FAI. In detail, 5° of forward PT decreases the acetabular version about 2.5°–5°, conversely increasing the femoral head coverage [8], while 10° of forward PT reduces the internal rotation in 90° of flexion about 5.9°, and up to 8.5° if the limb is 15° adducted [9]. The pro-FAI effect of forward PT is well known from a simple radiological examination of the acetabulum; the lateral center-edge angle and the percentage of acetabular crossover increase with pelvic forward tilt and decrease with back tilt [10]. Therefore, evaluating the pelvic sagittal rotation is of paramount importance before any conclusion about acetabular contribution to FAI is drawn from AP X-rays. Several cases of apparent pincer-FAI would be likely reclassified as normal if excessive forward PT was adequately recognized.

To what extent pelvic sagittal rotation influences acetabular orientation is explained by an individual anatomical angle, the acetabular tilt (AT), which measures the fixed acetabular rotation in respect of the pelvis [11]. The AT is the sagittal acute angle between the acetabular vertical axis or 180° meridian line (joining the center of rotation with the midpoint of the acetabular notch) and the anterior pelvic plane (APP, or reference plane defined by the two anterior superior iliac spines and by the pubic tubercles). Its normal value is 19° ± 6°, which means that the acetabulum is slightly back tilted with reference to the APP. The AT was demonstrated to be higher in dysplastic acetabula than in normal hips [12], thus possibly contributing to the characteristic anterolateral-deficient coverage associated with dysplasia. However, as FAI hips have not yet been assessed for AT, any hypotheses of lower angles are merely conjectural.

In addition to sagittal alignment, any possible frontal and axial pelvic malposition might asymmetrically affect acetabular orientation. In the case of axial rotation (i.e., scoliosis), the anterior socket would show an increased anteversion while the posterior socket would show a reduced anteversion [13], while in the case of pelvic obliquity (i.e., limb length discrepancy), the lower acetabulum would cover the femoral head more extensively than the higher one. Theoretically, both the posterior and inferior hips of these two scenarios would be more prone to impingement than the contralateral ones.

Since FAI pathomechanics is essentially determined by hip flexion, the sitting pelvic posture might be more important than the standing one. While sitting, the pelvis rotates backwards [14] in order to move the gravity line to the ischia, resulting in an SS close to 0, sometimes even negative.

The primary object of this systematic review is to analyze the relationship between pelvic posture (standing and/or sitting) and FAI to ascertain if peculiar pelvic postures may contribute to FAI (i.e., pelvic forward rotation, ipsilateral axial or frontal rotation) or if, conversely, FAI patients develop compensatory pelvic adaptations (i.e., pelvic backward rotation, contralateral axial or frontal rotation).

### Pelvic kinematics

The pelvic kinematics, i.e., the characteristics of pelvic motion in common ordinary life activities (walking, squatting, forward bending, stair climbing, etc.), is strictly influenced by the lumbo-pelvi-femoral rhythm, which is the synergistic relationship among lumbar flattening, pelvic posterior rotation and true hip flexion.

The mean pelvi-femoral ratio, or the ratio between pelvic rotation and overall thigh motion, is fairly steady regardless of the conditions of measurement—0.229 in the case of suspended bilateral active hip flexion [15], 0.181 in the case of unilateral active standing hip flexion [16], and 0.26–0.30, respectively in the case of unilateral/bilateral active supine hip flexion [17]. All these studies confirmed that pelvic rotation occurs throughout the whole hip flexion, accounting for approximately 20–25% of overall thigh flexion. Knee extension and inherent conditions of short hamstring increase the pelvis rotation due to the traction exerted by the tight hamstring through the ischial attachment [15].

Weightlifting may be performed by stooping or squatting. Stooping means to bend the trunk forward and requires not only hip flexion and pelvic back tilt, but also lumbar flexion; the lumbar-to-hip ratio was measured as 1.9, 0.9, and 0.4, respectively, in the early (0°–30°), middle (30°–60°) and late phase (60°–90°) of bending [18], with no significant differences between healthy subjects and low back pain patients. Thus, lumbar flexion prevails over hip flexion at the beginning of the motion, while hip flexion prevails over lumbar flexion close to the completion of the gesture. On the other hand, either single- or double-leg squat requires forward PT to compensate the posterior displacement of the pelvis due to knee flexion. In single-leg squat at peak knee flexion the pelvis rotates anteriorly by 26.77° and 30.19° on average in females and males, respectively [19].

One might suppose that subjects with low pelvi-femoral ratio in hip flexion, or with low lumbar-to-hip ratio in forward bending, or lastly with more anterior pelvic rotation in deep squat, would be more prone to FAI than subjects with higher ratios and less squat-related pelvic anterior rotation. Conversely, FAI patients might develop specific adaptations to raise those ratios and reduce pelvic anterior tilt while squatting in order to limit femoroacetabular engagement.

The second aim of this systematic review is to analyze the relationship between pelvic kinematics and FAI to ascertain if peculiar pelvic patterns of rotation may contribute to FAI or if, conversely, FAI patients develop compensatory patterns of pelvic motion.

The review was performed according to the PRISMA statement [20].

## Materials and methods

All the research studies that measured pelvic or spinopelvic positional and/or kinematic data in humans (patients or cadaveric specimens) affected by FAI were considered eligible, with or without controls. No limitations were set with regard to date or language of publication. Foreign articles would have been translated. Only articles whose full text was accessible were included.

PubMed/MEDLINE, Embase, Google Scholar and the Cochrane Library were initially searched on December 2014. Since the submission was delayed, the search was updated in August 2016, collecting the results from January 2015 onwards. Reference lists of selected records were analyzed to identify further eligible papers.

PubMed was searched using both MeSH terms and keywords, in order to retrieve the most recent articles. The most common index terms related to pelvic parameters, lumbopelvic rhythm, spinopelvic balance, pelvic posture and range of motion (ROM) were connected with FAI with the Boolean operator “AND” and 191 records were initially listed. Embase was similarly searched and 61 records were retrieved. A limited search was then conducted on Google Scholar, using the two most common keywords found in the records previously identified (‘spinopelvic’ and ‘femoroacetabular impingement’), and 18 items were found after exclusion of patents and citations. Lastly, the Cochrane Library was searched using the broadest criteria to identify all the Cochrane Reviews about FAI, and one record was retrieved. The full electronic search strategy of the four databases is presented in ‘Appendix 1’. After deduplication of the 271 records, 240 papers were identified. An additional search strategy was a manual review of the reference lists of all included articles. Further relevant papers known by the author would have been considered even if not resulted by the above search strategy. In 2016, an up-to-date query led to another 62 records from PubMed, 21 from Google Scholar, 21 from Embase, and none from Cochrane Reviews.

A data collection sheet was built to record all the relevant data reported by the included studies—title, authors, year of publication, level of evidence, materials (i.e., characteristics of the sample), methods of investigation, pelvic positional and kinematic parameters (PI, PT, SS, lumbar lordosis, pelvic ROM in the sagittal, axial and frontal plane). PT and SS were not measured in the selected studies, but substituted in one paper by other positional pelvic parameters (pelvic angle and pelvic inclination), that were added to the data collection sheet. Maximum anterior PT (as measured with motion capture analysis) and maximum squat depth were added to the sheet, since two studies provided these data items.

The risk of individual bias within studies was assessed, focusing on blindness of the investigators, power analysis, intra-/inter-rater reliability of measurements and on selective/incomplete data presentation, since most studies would have been observational and bias generation from intervention-related factors (i.e., random allocation and concealment, percentage of lost-to-follow up, etc.) would have not been applicable.

If homogeneous data were provided by two or more studies, a meta-analysis would have been performed and the risk of bias across studies would have been evaluated.

## Results

**Fig. 2:**
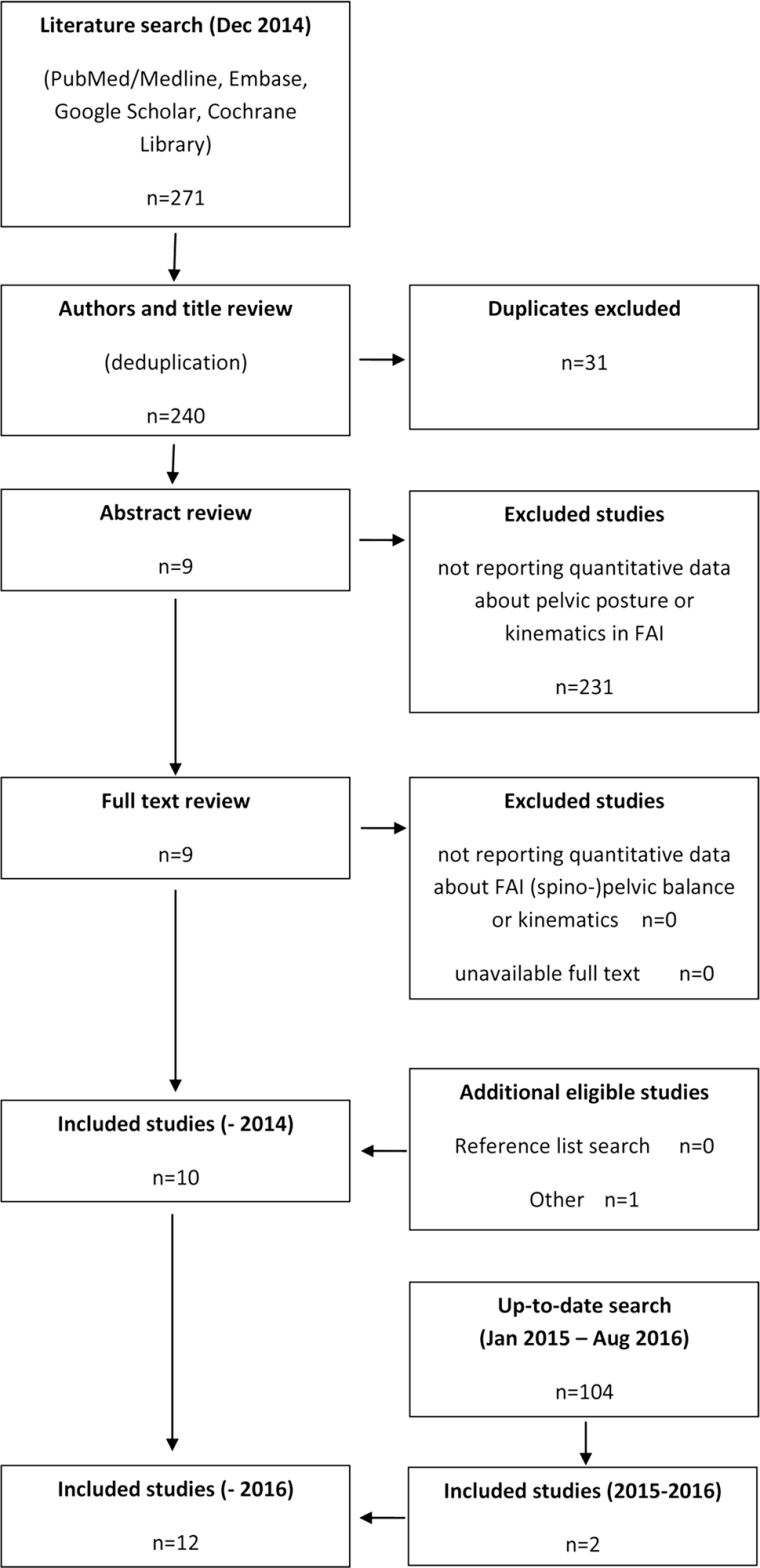
PRISMA flow diagram of study selection

All 240 papers from the first investigation were screened through title and abstract analysis; articles not providing quantitative data about pelvic or spinopelvic posture and/or ROM in FAI were excluded. Ten items were included (eight articles and two conference abstracts). The full text was available for all the articles. Nine items originated from database searches, one was added per author’s knowledge [21], and none per reference lists review (Fig. 2). An up-to-date search provided another 104 items, that after deduplication and review decreased to two papers only [22, 23].

Since the studies differ remarkably from each other regarding objectives and methods, they are grouped according to the main field of investigation (Table 1). The full data collection sheet is available as Online Resource 1.

**Table 1:** Synoptic table of the results

Field of investigation	Subfield of investigation	Study	Methods	Main findings	Main limitations
Pelvic posture	Pelvic incidence	Gebhart et al. [24]	Photography and manual goniometry	PI is lower in cam- and pincer-FAI than in normal hips	Only male cadaveric specimens; poor diagnostic criteria for pincer-FAI
		Hellman et al. [25]	Radiology	Symptomatic pincer and combined FAI have lower PI than healthy hips and pure cam-FAI	Historical healthy controls
		Weinberg et al. [22]	Radiology	Mixed-FAI have lower PI than controls	Retrospective CT review, without most clinical information
	Pelvic posture in acetabular dysplasia with cam deformity	Ida et al. [28]	Radiology	The presence of cam deformity increases the forward PT among dysplastic hips (only in upright position)	PI not measured
Pelvic kinematics	Hip flexion without weight-bearing	Van Houcke et al. [29]	Motion capture analysis	Higher pelvic back tilt with supine hip flexion in cam-FAI patients compared to healthy controls (only with active motion)	Blinding and intra-/inter-rater reliability not mentioned
	Walking and stair climbing	Kennedy et al. [30]	Motion capture analysis	Cam-FAI patients show less frontal pelvic ROM than healthy controls in level walking. No difference of axial and sagittal ROM	Blinding and power analysis not mentioned. No ROM exact values reported
		Rylander et al. [31]	Motion capture analysis	Pincer- and mixed-FAI patients display higher pelvic forward tilt and axial ROM while climbing stairs than healthy controls, both before and after surgery. No difference in level walking	No physical or radiological examination of healthy controls. Blinding and intra-/inter-rater reliability not mentioned
	Squat	Lamontagne et al. [32]	Motion capture analysis	Cam-FAI patients squat higher than control, with lower sagittal pelvic ROM and more pelvic forward tilt at maximum depth	Blinding and intra-/inter-rater reliability not mentioned
		Lamontagne et al. [33]	Motion capture analysis	Cam-FAI patients squat lower after corrective surgery, but sagittal pelvic ROM is not improved	Blinding and intra-/inter-rater reliability not mentioned. No ROM exact values reported
		Ng et al. [34]	Motion capture analysis	Low sagittal pelvic ROM is a crucial feature (along with α angle and neck-shaft angle) to determine symptoms in cam-FAI patients	–
		Wilson et al. [21]	Motion capture analysis	FAI patients squat lower if knee separation is allowed	Exact FAI type not reported. Blinding and intra-/inter-rater reliability not mentioned
		Bagwell et al. [23]	Motion capture analysis and force plate	Cam-FAI patients squat higher than controls but with less posterior PT, likely because the extensor moment is reduced. Reduced hip internal rotation	No blinding mentioned

### Pelvic incidence

Three studies [22, 24, 25] focused on the difference in PI between FAI hips and normal hips.

Gebhart et al. [24] evaluated 40 cadaveric pelves (80 hips) with photography and manual goniometry and compared PI between hips showing cam- or pincer-related bony abnormalities and hips without those abnormalities. They found that PI was significantly lower in both patterns of FAI compared with controls—43.1° ± 8.6° in 40 cam-FAI hips versus 47.7° ± 9.3° in 40 control hips (*p* = 0.02), and 42.5° ± 8.5° in 28 pincer-FAI hips versus 47.0° ± 9.2° in 52 control hips (*p* = 0.04). Obviously, since cadaveric specimens are studied, no information is available about hip symptoms, and FAI is diagnosed only from predisposing bony abnormalities. No female specimens were included, thus the findings might be gender-related. Moreover, the definition of pincer-FAI as acetabular anteversion <15° in the central transverse section perpendicular to the APP might be considered inadequate to recognize pure cranial retroversion, that may be underestimated in the central third of the socket, or global overcoverage (coxa profunda). Thus, some acetabula showing a strictly superior or superolateral overcoverage might be misdiagnosed as normal, as well as some coxae profundae that present a normal central anteversion. No blinding is mentioned, but inter-observer and intra-observer reliability is favorably assessed. Noticeably, the interpretation of the main finding is questionable, as the authors state that the lower PI would force the subjects to develop a forward PT (i.e., lower PT) determining a functional anterolateral overcoverage. Actually, if PI is low, both PT and SS are low (as PI = PT + SS), but the effects on acetabular rotation (and then on acetabular coverage) of these two positional variables are opposite—the lower the PT, the higher the anterolateral coverage; the lower the SS, the lower the anterolateral coverage. Whether PT or SS is more important is not yet established. The only relevant element is provided by Mac-Thiong et al. [26], who demonstrated that the correlation between PI and SS is stronger than between PI and PT, with SS accounting for 76% of PI on average and PT for just 24%. In other words, SS would decrease more than PT in the case of lower PI, possibly determining a lower anterolateral acetabular coverage, instead of the higher coverage supposed by the authors. However, it is more important to consider that low-PI pelves have lower sagittal ROM, and this could result in reduced back tilt in dynamic conditions that combine hip flexion and upholding the spinopelvic balance, with potentially enhanced femoroacetabular engagement (Table 1).

Hellman et al. [25] retrospectively evaluated PI using X-rays and computed tomography (CT) scans in 50 patients (60 hips) who underwent arthroscopy for FAI-related labral tear, and found that PI was on average lower in patients than in historical healthy controls (50.8° ± 11.3° vs 55.0° ± 10.6°, data obtained by Vialle et al. [27]). Within the patient sample, pincer-FAI showed lower PI than non-pincer-FAI; on the contrary, cam-FAI did not show different PI than non-cam-FAI. Methods presentation lacks information about blinding, number of examiners and measurement reliability, CT plane of acetabular version measurement, and adequacy of the AP pelvic view. The absence of true controls cannot be underestimated. However, favorably, the pincer-FAI definition looks more reliable than in the previous study, as multiple measurements are taken into account (acetabular index, center-edge angle and anteversion), and the analysis is limited to symptomatic patients. In conclusion, even though it is difficult to estimate the methodological quality due to the text limitations of this conference abstract, the finding is consistent with the first study, and further specifies that symptomatic pincer and combined FAI display lower PI than healthy hips and pure cam-FAI.

Lastly Weinberg et al. [22] retrospectively compared the CT images of 65 FAI patients and of 27 matched controls and found that mixed-FAI pelves displayed a PI significantly lower than controls (on average, 46.7° vs 57.1°). Pure cam and pincer deformities exhibited intermediate, non-significant values. The retrospective nature of the study, with no clinical information, and the definition of pincer deformity as retroverted socket (that might not identify cases of global pincer or coxa profunda) are the main limitations, while the reliability of measurements has been positively assessed.

### Pelvic posture in acetabular dysplasia and cam deformity

Ida et al. [28] used X-rays to evaluate the pelvic posture among cases of acetabular dysplasia (AD) (100 hips from 94 patients, mostly female) with (40 hips) and without (60 hips) cam deformity, and found that the pelves with combined AD and cam-FAI showed higher forward pelvic rotation (i.e., lower PT) while standing than pelves with pure dysplasia. In detail, the authors measured two less common pelvic parameters, the pelvic inclination angle (i.e., the acute sagittal angle between the line joining the promontorium to the upper surface of the pubic symphisis and the vertical axis) and the pelvic angle (i.e., the sagittal acute angle between the line joining the posterior edge of the sacral plate to the midpoint of the bicoxofemoral axis and the vertical axis), and found that both these parameters were significantly reduced when a cam deformity was associated with dysplasia, only in the upright position and not in decubitus. Notwithstanding the different SS, lumbar lordosis did not differ between the two groups. Intra-/inter-rater reliability was properly assessed, examiners were adequately blind regarding clinical information, and the two groups were comparable with regard to the most relevant confounding variables. Unfortunately no information is provided about PI. The authors conclude that cam deformity is associated with significant forward pelvic rotation in dysplastic acetabula, and this might affect the outcome of corrective acetabular surgery, predisposing to postoperative FAI.

### Pelvifemoral rhythm in hip flexion

The pelvifemoral rhythm differences between 17 cam-FAI patients (19 hips) and 12 healthy controls (24 hips) were assessed in a study conducted with an electromagnetic tracking device by Van Houcke et al. [29]. The patients exhibited a mean posterior pelvic rotation of 12.5° in supine active unilateral hip flexion, while controls had a mean posterior pelvic rotation of 9.1° (*p* < 0.001). No significant differences were measured in cases of supine passive flexion. Noticeably the pelvifemoral ratio was smaller than reported in other studies [15–17], approximately 8% in controls and 12% in patients actively flexing the hip, but the difference could be attributed to unilateral limb motion (that would elicit less pelvic back tilt than bilateral motion), to the deep knee flexion (that relaxes the hamstring) and to the peculiar contralateral positioning of pelvic markers (meant to reduce the effect of the skin shift). The study quality is good, with well-matched groups and adequate sample size, although no blinding or intra-/inter-rater reliability assessment is mentioned.

### Pelvic kinematics of walking and stair climbing

Kennedy et al. [30] explored hip and pelvis kinematics in level walking using three-dimensional (3D) motion capture analysis with retroreflective markers and compared 17 unilateral cam-FAI patients with 14 matched controls (case−control study). No power analysis or blinding is reported. Of the three planes of pelvic rotation, only frontal rotation was significantly diminished in the patient group (*p* = 0.004), but no exact values are provided for any of the above ROMs. The authors interpret this pattern, together with limited hip motion, as a different stabilization strategy developed by FAI patients in an activity that should not determine any true femoroacetabular engagement.

Rylander et al. [31] studied hip and pelvis kinematics in level gait and stair climbing, comparing the preoperative results of 17 unilateral pincer- or mixed-FAI patients with their postoperative results and with a group of 17 healthy controls, using a motion capture system. Regarding pelvic kinematics, they measured axial rotation and maximum anterior PT, and found that while level walking did not show any differences among the three groups, stair climbing showed significantly higher axial rotation and higher maximum forward PT in the FAI group (both before and after surgery) than in the control group. This specific pattern of pelvic motion is interpreted as a compensatory mechanism to save some hip internal rotation and extension, both possibly painful in the impinging hip. Indeed the same study demonstrated that hip sagittal ROM and hip internal rotation were reduced in FAI patients. Hip internal rotation in stair climbing obviously facilitates femoroacetabular engagement, while hip extension is probably avoided as a nonspecific source of pain, although pelvic extension might facilitate contralateral FAI. However, the authors do not specify when peak pelvic extension was measured during the gait cycle, and any comments are merely conjectural. With regard to possible bias, the study was adequately powered and controls were favorably matched, but their self-reported absence of hip problems was not confirmed by any physical or radiological examinations to rule out asymptomatic FAI. Lastly, measurement reliability was not assessed, and examiners were not reported to be blinded.

### Pelvic kinematics of squat

Three studies from the same group of investigators (University of Ottawa) [32–34] studied the pelvic kinematics of FAI patients and healthy controls while squatting. All these studies were performed with a 3D motion analysis system equipped with retroreflective markers.

The first study [32] compared 15 cam-FAI patients and 11 controls and found that the pelvic sagittal ROM was lower among cam-FAI patients than among controls, regardless of squat depth—14.7° ± 8.4° versus 24.2° ± 6.8° (*p* = 0.005). Moreover, cam-FAI patients could not squat as low as controls, reaching on average 41.5% of leg length versus 32.3% reached by controls (*p* = 0.037). PT change over the squat cycle turned out to be biphasic, determining an M-shaped line with two peaks and a trough. Peaks (i.e., maximum forward tilt) occur in the middle of each ascent and descent phase of the squat cycle, while the trough (i.e., maximum back tilt) occurs at maximum squat depth, when the motion reverses. Interestingly, while peaks are mostly similar between patients and controls, the trough is rather different. Healthy subjects have a deep trough, with the pelvis back tilted with respect to the start upright position; on the contrary FAI patients show a higher trough, thus preserving a forward tilted pelvis. This might facilitate femoroacetabular engagement at maximum squat depth. The study is conducted with adequate power analysis and case−control matching, although no blinding of the examiners or intra-/inter-observer agreement assessment is mentioned.

The second study [33] compared the pre- and postoperative condition of ten patients who underwent open corrective surgery for cam-FAI. Maximum squat depth was significantly improved by surgery from 36.9 to 33.2% of leg length on average (*p* = 0.027), while sagittal pelvic ROM was not. However, the small sample size and the heterogeneous timing of postoperative assessment might have contributed to this unexpected finding. In fact, the post hoc power analysis was adequately performed with squat depth as a key dependent variable. Blinding and intra-/inter-observer reliability are not mentioned. Although the authors do not provide exact sagittal pelvic ROM, the diagram pelvic pitch-squat cycle shows that operated patients have lower anterior PT over the whole gesture, except in the start/end upright position, when pelvic pitch is almost identical.

The third study [34] compared 12 symptomatic cam-FAI patients, 17 asymptomatic cam-FAI subjects and 14 healthy controls. Assignment of volunteers to the group of asymptomatic subjects or to the group of healthy controls was properly blinded. Sample size was adequately assessed, as well as inter- and intra-observer reliability. A stepwise discriminant function analysis revealed that the three most important variables to classify patients into one of the three groups (and to determine symptoms) are the radial α angle, the femoral neck-shaft angle and the sagittal pelvic ROM. In detail, controls could squat lower but had similar pelvic ROM to asymptomatic patients, who in turn could squat lower and showed wider ROM than symptomatic patients, but the differences were statistically insignificant.

A fourth study by Wilson et al. [21] is reported in a congress abstract. The authors used a motion capture system to analyze the kinematic differences between constrained and unconstrained squat (the former not allowing to increase the knees distance over the gesture, thus preventing from possible compensatory hip abduction and external rotation in FAI patients) in a series of 14 patients with an unspecified type of FAI. Regarding pelvic kinematics, the authors found that unconstrained squats reached lower heights than constrained squats (46.0 ± 15.1 vs 60.2 ± 12.8% of the sacral marker stance height, *p* < 0.001), confirming the effectiveness of hip abduction and external rotation to reduce femoroacetabular engagement, while frontal pelvic ROM was measured about 10.9° (unconstrained gesture) and 12.3° (constrained gesture), without a statistically significant difference between the two modalities. The authors believe this lateral inclination may depend on higher leaning on the dominant side. Unfortunately no information was recorded about sagittal or transverse ROM, and the text limits of abstract presentation make such papers lack several methodological standards, such as exact diagnosis (cam, pincer or combined FAI) and measurement reliability.

Data from the first three papers about squat biomechanics [32–34] were not combined due to methodological concerns, since most patients in the second study also belonged to the first study, and it is not clear whether some of the patients in the third study also belonged to the previous studies. In other words, a meta-analysis might simply duplicate data without adding truly novel information. All the other studies are simply too heterogeneous to allow a meta-analysis.

Recently, Bagwell et al. [23] confirmed all the previous findings. By comparing 15 cam-FAI patients with 15 controls using motion capture while squatting as low as possible, the authors could ascertain a reduced depth, an insufficient pelvic posterior tilt during the descent phase, a reduced extensor moment (that might justify the deficit of back tilt), and lower femoral internal rotation. Cases and controls look well matched and measurement reliability is favorably assessed.

## Discussion

The studies found by this systematic review provide a relatively novel perspective on the pathomechanics of FAI. In detail:

1. FAI-associated pelves seem to have a lower PI than controls, and such an anatomical feature is expected to reduce the maximum pelvic back tilt available, possibly enhancing femoroacetabular engagement in dynamic conditions that combine hip flexion and maintenance of the spinopelvic balance.
2. Dysplastic acetabula associated with cam deformity of the proximal femur exhibit higher pelvic forward tilt than dysplastic acetabula without such deformity. Care should be taken to avoid post-surgical FAI due to isolated acetabular correction.
3. Hip active flexion (but not passive) in the supine position determines more pelvic back tilt in cam-FAI patients than in controls, possibly due to a compensatory pattern of pelvic motion strictly related to muscle activation. It is to be noted that these results are not in contrast to point 1, as active flexion here is studied in the supine position, and PI (by the way, not measured) influences sagittal pelvic ROM only as far as balance upholding is concerned.
4. Level walking does not show different pelvic kinematics in FAI patients except a lower frontal ROM, that might depend on a different stabilization strategy poorly connected with FAI mechanism, since femoroacetabular engagement is unlikely to occur with level gait.
5. Stair climbing shows a higher peak of forward pelvic rotation and a wider range of axial pelvic rotation, almost certainly as part of a compensatory pelvic mechanism adopted to reduce hip internal rotation and extension, both sources of hip pain.
6. Squat biomechanics differ significantly between cam-FAI patients and controls; the former exhibiting less pelvic sagittal rotation (in accordance with point 1) and squatting higher, especially if cheating with knee separation is not allowed. The reduced sagittal back tilt keeps FAI-associated pelves forward tilted even in maximum squat depth, when femoroacetabular engagement is more likely to occur. Surgical correction of cam deformity allows deeper squat, but does not significantly affect the pelvic kinematics. Remarkably, sagittal pelvic ROM is found to be a relevant variable to determine whether a hip affected by cam deformity will be symptomatic or not. Unfortunately squat biomechanics of pincer-FAI has not yet been explored.

The present systematic review has significant limitations. First, no high-quality studies (level 1–2) were found about the subject. Thus, all the collected evidence is generated purely by case series and case−control studies, mostly with small sample sizes, and often without adequate blinding of the examiners. Second, the identified works are extremely heterogeneous regarding FAI diagnostic criteria, measured variables, and method of investigation (radiology or motion capture analysis), making it impossible to combine data in a reliable quantitative synthesis. Third, some data could not be accessed, since they were not published or could not be obtained by the authors.

Despite the above limitations, the qualitative findings of this review are important. Pelvic posture and kinematics seems to play a relevant role in FAI. The patients, especially if symptomatic, show a paradoxical lack of pelvic back tilt in standing hip flexions, i.e., in squatting, which enhances femoroacetabular engagement. Such an aberrant pattern might depend on a lower PI, but might also depend on insufficient extensor moment exerted by gluteus maximus and/or ischiocrural muscles. On the contrary, active hip flexion in decubitus elicits a compensatory, more pronounced back tilt to facilitate hip flexion without impingement. Level gait seems to be poorly affected, while stair climbing shows a compensatory pattern of augmented pelvic axial rotation and augmented peak forward tilt to reduce the most painful hip motions, namely internal rotation and extension. In other words, pelvic posture and kinematics in FAI are sometimes an expression of compensatory mechanisms, developed to reduce pain and discomfort, and sometimes an expression of paradoxical patterns that further enhance the impingement pathomechanism.

Higher quality evidence is needed to confirm these conclusions. Future research should focus on determining the anatomical sagittal rotation of the acetabulum with reference to the pelvis in normal and FAI hips, through the measurement of AT or similar morphologic parameters. The absolute multiplanar pelvic posture in FAI hips should be precisely evaluated, since knowledge of PI only is insufficient. Sitting posture should also be addressed, as it might be even more important for impingement than standing posture. Lastly, a modification of the paradoxical patterns of pelvic sagittal rotation might be attempted through dedicated physical therapy programs, and the assessment of their effectiveness might be the aim of future clinical research.

### Electronic supplementary material

Below is the link to the electronic supplementary material.

Supplementary material 1 (XLSX 14 kb)

## Appendix 1: full search strategy

*PubMed*: ((((hip AND impingement) OR FAI OR femoroacetabular impingement) AND ((lordosis OR lumbar spine OR spinopelvic balance OR (spine AND range of motion) OR (spine AND balance) OR (spine AND posture)) OR (pelvic incidence OR sacral slope OR pelvic tilt OR pelvic inclination OR (pelvis AND range of motion) OR (pelvis AND kinematics) OR (pelvis AND posture) OR lumbopelvic rhythm OR pelvifemoral rhythm OR lumbofemoral rhythm)))) OR ((impingement, femoracetabular [MeSH Terms] OR femoracetabular impingement [MeSH Terms] OR femoracetabular impingements [MeSH Terms]) AND ((balance, postural [MeSH Terms] OR lordosis [MeSH Terms] OR spine [MeSH Terms] OR lumbar vertebrae [MeSH Terms]) OR ((pelvis [MeSH Terms] OR pelvic bone [MeSH Terms] OR sacrum [MeSH Terms]) AND (posture [MeSH Terms] OR postural balance [MeSH Terms] OR postural equilibrium [MeSH Terms] OR postures [MeSH Terms] OR range of motion [MeSH Terms] OR motion [MeSH Terms] OR kinematics [MeSH Terms])))).

*Embase*: ((‘femoroacetabular impingement’/exp or ‘femoroacetabular impingement’ or ‘hip impingement’ and [embase]/lim) and (‘pelvis’ and ‘range of motion’)) or (‘pelvic tilt’ or ‘sacral slope’ or ‘pelvic incidence’ and (‘femoroacetabular impingement’/exp or ‘femoroacetabular impingement’ or ‘hip impingement’) and [embase]/lim).

*Google Scholar*: spinopelvic ‘femoroacetabular impingement’.

*Cochrane Library*: ‘femoroacetabular impingement’ OR (‘hip’ AND ‘impingement’):ti, ab, kw.
